# CYP2D6 Genotype and Primaquine Treatment in Patients with Malaria, Venezuela

**DOI:** 10.3201/eid3109.250316

**Published:** 2025-09

**Authors:** César Pacheco, Adán Hernández-Acosta, Narviz Pulido, Yvanna Ceballos, Daniel Saavedra, Cruz Gómez, Nancy Moreno, Flor Herrera

**Affiliations:** Centro de Investigación y de Estudios Avanzados del Instituto Politécnico Nacional, Mexico City, Mexico (C. Pacheco); Instituto de Investigación en Ciencias de la Salud de la Secretaría de Marina, Polígono Naval de San Pablo Tepetlapa, Mexico City (A. Hernández-Acosta); Universidad de Carabobo, Sede Aragua, Facultad de Ciencias de la Salud, Instituto de Investigaciones Biomédicas “Dr. Francisco J. Triana Alonso,” Estado Carabobo, Venezuela (N. Pulido, Y. Ceballos, D. Saavedra, N. Moreno, F. Herrera); FUNDASALUD Sucre, Dirección Estadal de Salud Ambiental, Sucre, Venezuela (C. Gómez)

**Keywords:** malaria, Plasmodium vivax, vector-borne infections, parasites, CYP2D6 metabolizer phenotype, primaquine, Venezuela

## Abstract

We determined CYP2D6*4 and CYP2D6* genotypes and metabolizer phenotypes in 96 patients with suspected malaria in Venezuela and found intermediate or poor metabolizer phenotypes in ≈25% of cases. Nine of 44 malaria patients had *Plasmodium vivax* recurrence. Public health authorities should evaluate the benefits of increasing total doses of primaquine for treatment.

Malaria is prevalent in different tropical and subtropical regions, including those in Africa, Asia, and Latin America (*1*). In Latin America, Brazil, Colombia, and Venezuela account for 76.8% of all reported cases. Most (72.1% in 2023) cases in that region are attributed to *Plasmodium vivax* (*1*). Sucre State is a malaria-endemic region in Venezuela, where *P. vivax* is almost the only species (*2*). Treatment of *P. vivax* malaria involves a combination therapy of chloroquine and primaquine, a prodrug that requires metabolic activation to elicit its antimalarial effect against hypnozoites (*3*).

Activation of primaquine is catalyzed by the metabolic enzyme cytochrome P450 2D6 (CYP2D6), which belongs to the CYP450 superfamily, a group of enzymes responsible for metabolism of many commonly prescribed drugs (*4*). The *CYP2D6* gene is highly polymorphic and has >150 different alleles (*4*), encoding CYP2D6 isoforms with normal, decreased, increased, or no activity. In Venezuela, several CYP2D6 genotypes exhibiting the most prevalent null allele, CYP2D6*4 (1846 G>A, rs3892097), and the less frequent CYP2D6*6 allele (1707delT, rs5030655) have been documented (*5*,*6*). For instance, the CYP2D6*4 allele was observed at a frequency of 14% in an urban admixed population from Aragua, a nonmalaria state, and in Amerindian populations at frequencies of 4.2%–42.5% from Zulia (nonmalaria state) and 1.7%–5.45% from Bolivar (malaria state). The CYP2D6*6 allele was observed at frequencies ranging from 0.3% to 1.2% in 2 urban admixed populations (*5*,*6*).

Malaria patients’ response to primaquine treatment is contingent on the level of CYP2D6 activity. In cases where CYP2D6 activity is low, probability of treatment failure is higher (*4*). Because CYP2D6 is necessary for primaquine metabolism, we determined the genotype of the most common CYP2D6*4 variant and a less common CYP2D6*6 variant with null activity and predicted the metabolizer phenotype in a sample of Mestizo persons with suspected *P. vivax* malaria from malaria-endemic Sucre state, Venezuela. We also evaluated the response to the standard treatment with primaquine.

## The Study

We conducted this study by using a sample of 96 patients (60 men and 36 women) exhibiting malaria symptoms. The patients were unrelated to each other and >18 years of age. We recruited the patients in December 2022 from 4 health centers in the city of Cumaná, municipality of Sucre, Sucre state, Venezuela (Figure). We collected peripheral blood samples from the patients after they provided informed consent as approved by the Instituto de Investigaciones Biomédicas, Universidad de Carabobo Bioethics Committee (approval no. CBIIB-UC/2022-2).

**Figure:**
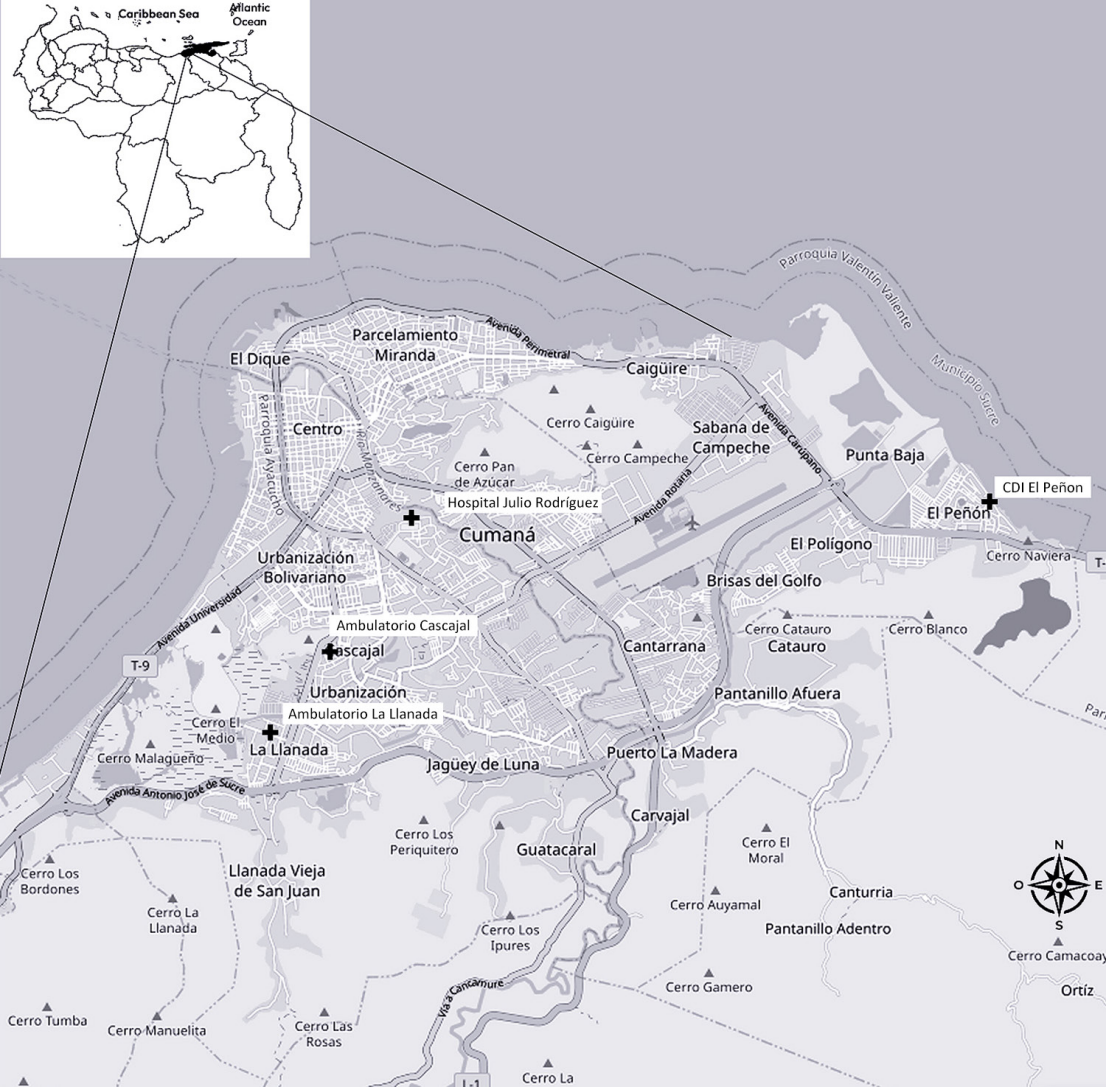
Geographic area of Sucre municipality from a study of CYP2D6 genotype and primaquine treatment in malaria, Venezuela. Crosses represent health center locations in the Sucre municipality. Inset shows map of Venezuela with Sucre state in black.

We performed microscopic malaria diagnosis and validated positive results by using PCR (*3*). We extracted genomic DNA as previously described (*7*) and conducted genotyping in accordance with previously outlined methods (*5*). If we observed no nucleotide change in the 2 allelic variants analyzed, we designated the allele as CYP2D6*1. 

We predicted metabolizer phenotype by using the activity score (AS) system (*8*). In brief, we assigned values to the identified alleles ranging from 0 for no-function alleles (*4, *6) to 2 for normal-function alleles (*1). The AS value of a given genotype is the sum of the assigned values for each allele. We designated patients with AS of 0 as poor metabolizers, AS of 1 as intermediate metabolizers, and AS of 2 as normal metabolizers (*8*,*9*).

Among 96 participants, 44 (45.83%) were malaria positive. As expected, *P. vivax* malaria was predominant; 43 (97.72%) of the 44 malaria-positive patients were infected with *P. vivax*, and 1 (2.27%) was infected with *P. falciparum*. Of the 44 malaria-positive patients, 30 (68.18%) were men and 14 (31.81%) were women, which we anticipated: men have a higher malaria risk than women because their occupations are more likely to involve outdoor work, like fishing. The patients received standard oral primaquine treatment (0.25 mg/kg/d for 14 days) (*3*). On day 28, we evaluated patients for malaria recurrence, which was characterized by fever and parasitemia (*10*).

We compared samples from the 52 malaria-negative participants and the 44 malaria-positive participants and found similar genotype profiles. CYP2D6*1*1 was the predominant genotype, which indicated a normal metabolizer phenotype. We saw a percentage of 75% in the 96 patients with the CYP2D6*1*1 genotype across both nonmalaria and malaria patients (Table). The other 25% of analyzed genotypes manifested as intermediate or poor metabolizer phenotypes, among which the CYP2D6*1*4 genotype showed the highest frequency and had an average of 17.75% across both groups. That frequency is substantially higher than the low frequency (2.37%) of that genotype identified within the population identified in Madagascar (*11*). However, in 5 areas of Brazil, the CYP2D6*1*4 genotype exhibited a high frequency (35%–50%) (*12*). That variation can be attributed to the different ethnic origins of the genotype, which differ across regions. The ancestors of the population of Madagascar are believed to be from Asia and Africa, whereas the ancestors of the highly heterogeneous populations from Venezuela and Brazil are thought to be from Spain and Portugal. In addition, the CYP2D6*4 genotype frequency within the group from Europe is high (*13*). The metabolizer phenotype profile showed notable similarity across both groups; 75% of patients exhibited a normal metabolizer profile, 20.8% showed an intermediate metabolizer profile, and 4.19% displayed a poor metabolizer profile. Those values agree with values observed in ethnicity from Europe, thereby providing further substantiation for the initial assertion (*13*).

**Table:** Genotypes CYP2D6*4 and CYP2D6*6 and predicted phenotypes malaria and non malaria in patients with suspected malaria, Venezuela

Genotype	Activity score	Predicted phenotype	Nonmalaria patients, no. (%), n = 52	Malaria patients, no. (%), n = 44
1*1	2	Normal metabolizer	39 (75)	33 (75)
1*4	1	Intermediate metabolizer	9 (17.31)	8 (18.19)
1*6	1	Intermediate metabolizer	2 (3.85)	1 (2.27)
4*4	0	Poor metabolizer	1 (1.92)	1 (2.27)
6*6	0	Poor metabolizer	0	1 (2.27)
6*4	0	Poor metabolizer	1 (1.92)	0

*P. vivax* recurrence was observed in 9 (20.5%) patients (6 men and 3 women); 7 exhibited an intermediate metabolizer phenotype, and 2 exhibited a poor metabolizer phenotype. That phenomenon might be attributed to a substantial correlation between the *CYP2D6* alleles with diminished activity, such as CYP2D6*4 and *6, and the occurrence of primaquine therapeutic failure in patients infected with *P. vivax* (*4*). High rates of relapses have already been reported in Venezuela (*14*; J. Huber et al., unpub. data, https://www.medrxiv.org/content/medrxiv/early/2022/05/12/2022.04.19.22274042.full). The recurrence of *P. vivax* shown in this study might be preventable by administering a higher dose (7.0 mg/kg) of primaquine instead of the conventional dose (3.5 mg/kg) (*10*).

The first limitation of this study is the low number of patients; thus, our results might not be representative of all malaria-infected populations. Second, we only conducted patient monitoring on day 28 and thus only determined the clinical and parasitologic response and not the absence of *P. vivax* recurrence, which requires meticulous monitoring over a period of ≈6 months (*9*); thus, we might have missed recurrence that occurred after 28 days.

## Conclusions

We estimated that ≈25% of malaria patients had nonfunctional alleles that would impair efficacy of primaquine. The corresponding predicted intermediate metabolizer phenotype was 20.8% and the poor metabolizer phenotype was 4.19% in a sample of malaria patients and nonmalaria patients susceptible to infection with *P. vivax* in Sucre, a malaria-endemic state of Venezuela. Those data are consistent with the 20.5% recurrence rate observed in *P. vivax* patients. To effectively treat malaria in the region, we recommend that public health authorities evaluate the potential benefits of increasing total doses of primaquine (7.0 mg/kg; 0.5 mg/kg/d) over a period of 14 days as an alternative to the current treatment regimen.
